# Chemical and Bioactive Characterization of the Essential Oils Obtained from Three Mediterranean Plants

**DOI:** 10.3390/molecules26247472

**Published:** 2021-12-10

**Authors:** Virginie Xavier, Tiane C. Finimundy, Sandrina A. Heleno, Joana S. Amaral, Ricardo C. Calhelha, Josiana Vaz, Tânia C. S. P. Pires, Irene Mediavilla, Luis Saúl Esteban, Isabel C. F. R. Ferreira, Lillian Barros

**Affiliations:** 1 Centro de Investigação de Montanha (CIMO), Instituto Politécnico de Bragança, Campus de Santa Apolónia, 5300-253 Bragança, Portugal; virginie.xavier@ipb.pt (V.X.); tiane@ipb.pt (T.C.F.); sheleno@ipb.pt (S.A.H.); jamaral@ipb.pt (J.S.A.); calhelha@ipb.pt (R.C.C.); josiana@ipb.pt (J.V.); tania.pires@ipb.pt (T.C.S.P.P.); iferreira@ipb.pt (I.C.F.R.F.); 2 REQUIMTE/LAQV, Faculdade de Farmácia, Universidade do Porto, Rua de Jorge Viterbo Ferreira, 228, 4050-313 Porto, Portugal; 3 CEDER-CIEMAT, Autovía de Navarra A-15, Salida 56, 42290 Lubia, Spain; irene.mediavilla@ciemat.es

**Keywords:** essential oils, shrubs, bioactivities

## Abstract

*Cupressus sempervirens* L., *Juniperus communis* L. and *Cistus ladanifer* L. are Mediterranean arboreal and shrub species that possess essential oils (EO) in their leaves and branches. This study aimed at characterizing the EOs obtained by steam distillation from the three species collected in different locations from Spain (Almazán, Andévalo, Barriomartín, Cerezal, Ermitas and Huéscar). For this purpose, volatiles composition was determined by GC-MS, and different bioactivities were evaluated. The highest content in terpenes was observed in *C. sempervirens* (Huéscar origin) followed by *J. communis* (Almazán origin), corresponding to 92% and 91.9% of total compounds, respectively. With exception of *C. ladanifer* from Cerezal that presented viridiflorol as the most abundant compound, all the three species presented in common the α-pinene as the major compound. The EOs from *C. ladanifer* showed high antibacterial potential, presenting MIC values from 0.3 to 1.25 mg/mL. Concerning other bioactivities, *C. ladanifer* EO revealed an oxidation inhibition of 83%, while *J. communis* showed cytotoxicity in the MCF-7 cell line, and *C. sempervirens* and *C. ladanifer* EOs exhibited the highest potential on NCI-H460 cell lines. Nevertheless, some EOs revealed toxicity against non-tumoral cells but generally presented a GI_50_ value higher than that of the tumor cell lines.

## 1. Introduction

For decades, the need to change from a fossil-based economy to bio-based systems has been discussed. It has been pushing the industry to transition towards a green, sustainable and circular economy, improving energy efficiency by using renewable raw materials [1,2]. In this sense, the use of lignocellulosic biomass, which represents 89.3% of the total biomass, is being explored as a potential substrate to obtain compounds of interest, such as bioactive molecules, through an integrated biorefinery approach.

Some crop and forest biomass resources can be an effective source of high-value compounds since this raw material is underused and naturally recycled into ecosystems. Thus, it is a low-cost feedstock that can assume a role of great importance for food, pharmaceutical and cosmetic industries due to beneficial effects, including antioxidant, antimicrobial, anti-inflammatory and anti-tumoral properties, that have been attributed to different compounds present in such agroforestry biomass [3,4].

The majority of the shrubs belonging to the Cupressaceae and Cistaceae families can be found all over the Mediterranean region and have the capacity of growing in open areas with deficient soils and harsh environments [5]. In addition, they are intensely aromatic plants due to the high content of essential oils (EOs) in their twigs and leaves [6]. In particular, the genus *Cupressus* and *Juniperus* (Cupressaceae) and *Cistus* (Cistaceae) are widespread include some species, such as *Cupressus sempervirens* L., *Cistus ladanifer* L. and *Juniperus communis* L., being their extracts or EOs used for many years in traditional medicine [7].

Moreover, bioactivity studies reported these species’ antimicrobial, anti-inflammatory, and antioxidant properties [8,9]. Several pharmacologically interesting compounds were already identified in their extracts, e.g., tannins, flavonoids, phenolic acids and terpenes [10,11]. Given their richness in bioactive molecules and bioactive power, these compounds, can effectively apply in different industries, such as food, cosmetics, and pharmaceuticals. [6,12].

The species *C. sempervirens* L. and *J. communis* L. are widely planted as ornamental shrubs in parks and gardens, while *C. ladanifer* L. is frequently found in wild areas. However, these species present a high potential to be grown in marginal lands, which are not used either for other agricultural or forestry purposes, allowing for improving soil fertility and organic carbon stocks while simultaneously generating biomass that can be used for bio-based value chains.

In the BBI-JU BeonNAT project, some of these species have been selected to create new dedicated plantations that can be used to produce valuable EOs. EOs are complex mixtures of hydrocarbons and oxygenated hydrocarbons from the isoprenoid pathways, mainly mono-, di-, and sesqui- terpenes [13]. So far, different studies have investigated the chemical composition of *C. ladanifer*, *C. sempervirens* and *J. communis* EOs by gas chromatography coupled to mass spectrometry (GC-MS) [14,15,16,17], with α-pinene being frequently reported as the major component in these three species [10].

However, they mainly refer to the essential oil obtained by laboratory hydrodistillation from the leaves of *C. ladanifer* and *C. sempervirens*, as also from *J. communis* berries with few or non-existing data regarding other plant parts. Hydro and steam distillations are some of the most traditional ways to isolate volatile compounds from medicinal and aromatic plants [18]. The extraction method is a recognized factor that may greatly impact the quality of EOs. Moreover, the chemical composition of essential oils can be affected by other factors, such as environmental conditions [19].

Therefore, as part of the BBI-JU BeonNAT project, the present study aimed at evaluating the chemical composition of the EOs extracted by steam distillation from the crown biomass (with branches diameter < 50 mm) of these three species and studying their bioactive properties, namely antioxidant, antibacterial, cytotoxic and anti-inflammatory, to access its potential as ingredients for bio-based products development in different industries. Moreover, each species was collected from two different locations in Spain to look for different chemotypes associated with different geographical locations and/or elevations.

## 2. Results and Discussion

### 2.1. Essential Oil Yields

**Figure 1:**
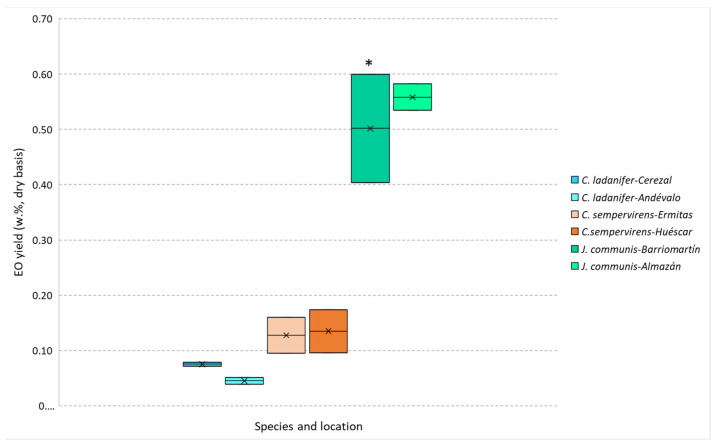
*J. communis*, *C. sempervirens* and *C. ladanifer* EOs yields obtained by steam distillation. * Samples differ significantly (*p* < 0.05) between the different origins, obtained by Student’s *t*-test.

The extraction yield of the EOs obtained by steam distillation was higher for *J. communis*, followed by *C. sempervirens* and *C. ladanifer*, as can be observed in Figure 1. The obtained values are within the range reported in the literature for *C. ladanifer* (from 0.01 to 0.63%) [20,21,22,23] while being lower for *C. sempervirens* for which reported yields varied from 0.20 to 0.87% [24,25,26] and higher for *J. communis* (reported yields from 0.05 to 0.70%) [23,27,28,29,30]. These differences are most probably related to (i) the used samples (crown biomass that includes twigs, leaves and fruits instead of leaves or berries); (ii) the location where the plant samples were collected; (iii) the date when these samples were obtained, and (iv) the EO extraction methodology. These factors are important since the extraction yield of essential oils depends on some variables like the part of the plant material, seasonal variations, environmental and cultivation conditions, plant age, harvesting time, and type of distillation [17].

### 2.2. Chemical Composition

The composition of the EOs in terms of volatile compounds is presented in Table 1. The GC-MS analysis led to the identification of different components, representing between 84–92% of total oil constituents. The EOs of the three plant species showed four main chemical classes with a predominance of monoterpene hydrocarbons in all samples, except for *C. ladanifer* EO from Cerezal, for which oxygen-containing sesquiterpenes was the leading group. In both samples of *J. communis*, EO sesquiterpenes are also an important fraction, as they ranged from 19.3% to 26.2%. In general, the major compounds identified in *C. ladanifer* were common in both Cerezal and Andévalo samples but in different amounts: viridiflorol (24.13 ± 0.74 and 13.36 ± 1.41%), α-pinene (19.27 ± 0.26 and 42.50 ± 0.96%), ledol (6.94 ± 0.36 and 4.06 ± 0.17%), bornyl acetate (5.01 ± 0.02 and 4.16 ± 0.10%) and camphene (6.66 ± 0.01 and 2.15 ± 0.07%). While α-pinene is the predominant compound in the sample from Andévalo, in the one from Cerezal the main compound was viridiflorol. The obtained data are comparable and in line with the available reports described by other authors, despite some studies describing lower amounts of α-pinene than the ones herein reported. According to Verdeguer, et al. [31], the aerial parts of *C. ladanifer* collected in Spain presented high percentages of trans-pinocarveol (20%), followed by viridiflorol (13.59%), bornyl acetate (7.03%), terpinen-4-ol (6.37%), 2(10)-pinen-3-one (5.05%), α-pinene (4.70%) and camphene (1.17%). This report mentioned that other studies have previously found α-pinene (39%), viridiflorol (11.8%), ledol (3.3%) and bornyl acetate (3.1%) as major compounds in leaves and stems of *C. ladanifer* plants of Spanish origin collected in Corsica. Low amounts of α-pinene but higher in viridiflorol were reported in *C. ladanifer* fresh leaves and small branches from Morocco, which showed viridiflorol (19%), bornyl acetate (17%), camphene (12%), ledol (8%) and α-pinene (5%) as the main compounds [32]. Mediavilla et al., 2021 which used the same equipment and same steam distillation conditions for samples collected in Spain in two different periods, found α-pinene (39–52%), viridiflorol (6–10%), bornyl acetate (3%) and camphene (2–3%) as the main compounds in *C. ladanifer* samples. Zidane described a slightly different profile, Elmiz et al. (2013), who studied *C. ladanifer* fruits, stems, flowers and leaves and reported camphene (15.5%), borneol (11.1%), cyclohexanol-2,2,6-trimethyl (7.3%), terpineol-4 (6.3%) and α-pinene (4.2%) as the major compounds.

Despite presenting some quantitative differences, the major compounds identified in the samples of *J. communis* were also similar in both locations. The major compounds in Barriomartín, Spain, were α-pinene (35.05 ± 0.02%), limonene (15.01 ± 0.26%), cis-thujopsene (8.04 ± 0.07%), sabinene (6.72 ± 0.40%), β-caryophyllene (3.51 ± 0.14%) and β-myrcene (3.24 ± 0.06%), while in Almazán, Spain, were α-pinene (23.96 ± 0.41%), limonene (21.30 ± 0.03%), cis-thujopsene (8.19 ± 0.14%), sabinene (7.86 ± 0.01%) and germacrene D (2.69 ± 0.03%). In general, the chemical composition was in good agreement with that of juniper berries essential oil defined in the European Pharmacopoeia (Ph. Eur. 10th), except for limonene (from 2–12%) [33]. Thus, these results suggest that juniper crown biomass can be a promising low-cost source of juniper EO due to the similar global composition with EO of juniper berries. Moreover, the obtained chemical profile is generally similar to that reported in previous studies regarding both juniper needles and berries. Chatzopoulou and Katsiotis [34] reported that the essential oil from the leaves of *J. communis* from northern Greece was dominated by α-pinene (41.3%) and sabinene (17.4%) while Cabral, et al. [35] and Caramiello, et al. [36] identified sabinene (30.5% and 41.4%, respectively) and α-pinene (29.6% and 13.4%, respectively) as major compounds in the needle oil from *J. communis* species grown in Turkey and the Northwestern Italian Alps, respectively. Mediavilla, Blázquez, Ruiz and Esteban [23] found α-pinene (16–21%), sabinene (18–34%), limonene (6–8%), β-myrcene (3–5%) and β-phellandrene (3–4%) as the major compounds in samples collected in Spain in two different periods. Höferl, Stoilova, Schmidt, Wanner, Jirovetz, Trifonova, Krastev and Krastanov [16] identified α-pinene (51.4%), myrcene (8.3%), sabinene (5.8%), limonene (5.1%) and β-pinene (5.0%) as the main compounds of *J. communis* berries while Koukos and Papadopoulou [37] detected α-pinene (27.22–62.08%), limonene (1.31–30.96%), myrcene (5.41–20.23%), sabinene (0.35–16.47%), citronellol (5.06–15.57%), β-caryophyllene (0.79–6.61%), borneol (0.86–4.51%) and β-pinene (1.89–3.47%). According to these authors, the minor compounds present in the fruit of *J. communis* were α-terpineol (0.50–2.64%), cedrol (0.56–1.84%), bornyl acetate (0.25–1.51%), eugenol (0.45–2.33%), geraniol (0.52–1.15%) and terpinolene (0.33–1.21%) [38]. These authors also found α-pinene (29.17%) and β-pinene (17.84%), sabinene (13.55%), limonene (5.52%), and myrcene (0.33%) as the main compounds present in the berries of this species. Therefore, from a qualitative point of view, the results obtained are in agreement with the available data on the literature.

Concerning the *C. sempervirens* species, the samples of the two evaluated locations in Spain presented similar qualitative and quantitative compositions. In both (Huéscar and Ermitas), the most abundant compound was α-pinene (52.32 ± 3.48 and 55.95 ± 0.46%, respectively) followed by 3-carene (16.18 ± 1.12 and 13.09 ± 2.70%) and cedrol (4.63 ± 0.25 and 2.88 ± 0.11%). Additionally, both locations presented amounts in the range of 2.5–5% for limonene (2.74 ± 0.23 and 2.66 ± 0.01%), germacrene D (3.13 ± 0.19 and 1.38 ± 0.01%) and terpinolene (3.58 ± 0.34 and 2.60 ± 0.02%), with samples from Huéscar presenting slightly higher amounts of these three compounds. The two locations also presented similar contents of other minor compounds, such as β-myrcene (2.63 ± 0.19 and 2.15 ± 0.07%), β-pinene (1.42 ± 0.13 and 1.25 ± 0.02%) and α-fenchene (0.64 ± 0.04 and 0.74 ± 0.03%). The compounds mentioned above are in good agreement with the ISO 20809 [33] concerning the Spanish type *C. sempervirens*, except for 3-carene from Ermitas location and cedrol from Huéscar location that fit the French type, and terpinolene that is not part of the chemical profile presented on ISO 20809 [33]. According to Selim, Adam, Hassan and Albalawi [24] α-pinene (48.6%), δ-3-carene (22.1%), limonene (4.6%) and α-terpinolene (4.5%) were the main components of the oil obtained from the aerials parts of *C. sempervirens* collected in Saudi Arabia. The same main compounds were found in the aerials parts of this species from Tunisia, being α-pinene (37.14%), δ-3-carene (19.67%), limonene (5.43%) and α-terpinolene (4.69%) as the most abundant compounds [25]. Mazari, et al. [39] found cedrol (8.3%) as the second most important constituent of the *C. sempervirens* oil. In another study, Rguez, Djébali, Ben Slimene, Abid, Hammemi, Chenenaoui, Bachkouel, Daami-Remadi, Ksouri and Hamrouni-Sellami [17] evaluated the importance of the phenological stages and found that the main constituents for all the stages were α-pinene, β-caryophyllene and germacrene D. Nevertheless, some differences between the phenological stages were noticed by the presence of specific minor compounds meaning that the vegetative stage was characterized by the appearance of γ-cadinene (2.75%) and γ-muurolene (2.95%), the flowering stage by camphene (0.33%), m-mentha-1,8 diene (0.32%) and α-farensene (0.21%) and the fructification stage was characterized by the presence of δ-3-carene.

### 2.3. Bioactive Evaluation

#### 2.3.1. Antibacterial Activity

Table 2 presents the results of the antibacterial capacity against a panel of bacteria selected according to their importance in public health. None of the essential oils showed the potential to inhibit the growth of gram-negative bacteria *K. pneumoniae* and *P. mirabilis*. On the other hand, the *E. coli* strain was sensitive to all the tested EOs. At the same time, *M. morganii* was only sensitive to *C. ladanifer* EOs, being notably inhibited by the EO from Cerezal location (MIC = MBC = 0.6 mg/mL). Among the samples, *C. ladanifer* EO was the only one that presented inhibitory potential against the gram-negative bacteria *P. aeruginosa,* which is frequently associated with nosocomial infections.

In general, gram-positive bacteria presented lower MIC values, being most susceptible compared with the gram-negative strains. *E. faecalis* was inhibited and killed by all the tested EOs, with MIC and MBC values ranging from 0.6 mg/mL to 2.5 mg/mL. The only sample that could not inhibit the growth of all the tested gram-positive bacteria was *J. communis* EO from Barriomartín location as it was not effective against *L. monocytogenes*.

In general, *C. ladanifer* exhibited the most potent antimicrobial potential, with samples from both locations showing the lowest MIC and MBC values for both gram-negative and gram-positive bacteria compared with other data described in the literature. Outstanding results were obtained against MRSA, a bacteria associated with nosocomial infections. According to Mohammed, Said, Fouzia, Kawtar, Zoubida, Abdelilah, Elhourri and Ghizlane [21], *S. aureus* shows high sensitivity (MIC = MBC = 6.25 mg/mL), and *E. coli* and *P. aeruginosa* respond very positively (MIC = MBC = 25 mg/mL for both strains) to the essential oil obtained from *C. ladanifer* stems and leaves. Curiously they found different compounds in major contents in the chemical characterization, suggesting that synergism between these compounds can occur. Identical results were reported by Benali, et al. [40] against *S. aureus* (MIC = MBC = 6.25 mg/mL), with much lower MIC and MBC values being observed for *P. mirabilis* (MIC = MBC = 0.19 mg/mL), which was the most sensitive strain when assessing the antimicrobial activity of *C. ladanifer* EO (aerial parts).

On the contrary, using a different method, the disk agar diffusion method, Tavares, Martins, Faleiro, Miguel, Duarte, Gameiro, Roseiro and Figueiredo [22] reported a weak antimicrobial activity for *C. ladanifer* essential oil against *E. coli* and *S. aureus*. Benayad, et al. [41] also studied the antimicrobial effect of *C. ladanifer* EO (full plant) and reported MIC values between 50–500 µg/mL, with the lower MIC being obtained against multiresistant *S. aureus* (MIC = 50 µg/mL). Although Benayad, Mennane, Charof, Hakiki and Mosaddak [41] obtained stronger activity with lower MIC values, they also verified that the EO was effective against both gram-positive and gram-negative bacteria, with better activity against the gram-positive, and no inhibition was observed for *K. pneumoniae* at the higher tested concentration. Although the studies reported by Benali, Bouyahya, Habbadi, Zengin, Khabbach, Achbani and Hammani [40] and Benayad, Mennane, Charof, Hakiki and Mosaddak [41] are in good agreement with the present ones, these authors did not analyze the chemical composition of the EO; thus it was not possible to corroborate the results with the chemical composition.

Regarding *C. sempervirens* EO, different studies reported its inability to inhibit the growth of *P. aeruginosa*, either using the agar dilution [42] or the broth microdilution [43] methodologies. Hammer, Carson and Riley [43] achieved identical results for *P. aeruginosa*; however, the oil obtained from the leaves and twigs was able to inhibit the growth of *E. coli*, *S. aureus* and *E. faecalis* at the maximal concentration tested (MICs > 2.0% *v/v*) which is in line with the herein obtained results. Similar results were obtained by [44], who reported that the EO from the aerial parts of *C. sempervirens* showed an antimicrobial activity more pronounced against gram-positive than gram-negative bacteria, with MIC and MBC values of 0.07 µg/mL and 0.31 µg/mL for *S. aureus* and *E. coli* respectively. One of the main compounds identified was δ-3-carene in both studies. Nevertheless, in this study the authors also reported that *P. aeruginosa* was sensitive to *C. sempervirens* EO (MIC = MBC= 0.31 µg/mL). Contrarily to previous studies and the results herein obtained, Mazari, Bendimerad, Bekhechi and Fernandez [39] reported that the EO from *C. sempervirens* (leaves) was ineffective against *S. aureus*, E. faecalis, *E. coli* and *P. aeruginosa* at the highest concentration tested (10 µL/mL). In this study, the common major compound (carene) was not identified, which probably justifies these results.

Regarding the results herein obtained for *J. communis* EO, they are in good agreement with previous studies that also reported the capacity of the EO obtained from different parts of the plant against *S. aureus*, *E. faecalis* and *E. coli* while not presenting antimicrobial activity at the highest tested concentration (2% *v/v* in the broth dilution and 5 mg/mL in the agar disc diffusion methods) for *K. pneumoniae*, *P. mirabilis* and *P. aeruginosa* [6,43]. Angioni, Barra, Russo, Coroneo, Dessí and Cabras [27] reported contradictory results, which showed that the antimicrobial activity of the *J. communis* EO from berries and leaves was generally nonsignificant against *S. aureus* and *E. coli* at the highest concentration tested (900 µg/mL). Falcão, et al. [45] evaluated the antimicrobial activity of two commercial samples of *J. communis* EO and one obtained by hydrodistillation of the berries and found that all were able to inhibit the growth of *S. aureus*, *Bacillus cereus*, *Bacillus subtilis*, *E. coli*, *E. faecalis* and *K. pneumonia*. In contrast, only one commercial EO presented activity against *P. mirabilis*, *P. aeruginosa* and *Salmonella Typhimurium*. The wider range of activity of this EO was related to its different chemical composition compared to the other samples, namely its higher content in oxygenated monoterpenes, such as terpinene-4-ol and 1,8-cineole, which have been associated with antimicrobial properties. The herein studied *J. communis* EO present very low content of terpinene-4-ol and 1,8-cineol was not detected, what can possibly explain the lower activity of these oils.

Comparing the antibacterial potential of the analyzed EOs with common antibiotics, none of them could compete with these commercial drugs. Nevertheless, commercial drugs are isolated compounds, while EOs are a mixture of different compounds. Nonetheless, given the growing resistance to antibiotics, new antibacterial agents are needed, and the exploitation of novel sources of antibacterials is a major research topic worldwide.

#### 2.3.2. Antioxidant Activity

Analyzing the antioxidant values obtained from the cellular-based assays (Table 3), all the samples revealed the capacity to inhibit the oxidation process, highlighting the sample *J. communis* from Almazán that inhibited about 78% of oxidation, presenting a GI_50_ value of 324 ± 8 µg/mL, and *C. ladanifer* from Andévalo that inhibited about 83% of oxidation and exhibited a GI_50_ of 336 ± 8 µg/mL.

To the best of our knowledge, data are scarce in the literature regarding the antioxidant properties of the studied EO, with most studies available relying on the use of the DPPH method. Boukhris, Regane, Yangui, Sayadi and Bouaziz [44] measured the antioxidant activity of *C. sempervirens* EO by the radicals-scavenging effect on 2,2-diphenyl-1-picrylhydrazyl (DPPH) and reported an EC_50_ value of 7.70 ± 0.70 µg/mL, however, higher IC_50_ values (151 µg/mL and 290.09 µg/mL) have been reported for this species EO using the same methodology [25,26]. Additionally, using the DPPH assay, Höferl, Stoilova, Schmidt, Wanner, Jirovetz, Trifonova, Krastev and Krastanov [16] reported an IC_50_ of 944 µg/mL for Juniper berry oil. On the contrary, using this assay, no activity was found for *C. ladanifer* EO at the highest tested concentration (100 µg/mL) while a value of 0.1 ± 0.06 AAE/g was reported using the Ferric Reducing Antioxidant Power (FRAP) methodology.

#### 2.3.3. Cytotoxic and Anti-Inflammatory Activity

The effects of the oils obtained by steam distillation on the growth of four human tumor cell lines (MCF-7, NCI-H460, CaCo2 and AGS) and two non-tumoral cell lines (Vero and PLP2), represented as the concentration that caused 50% of cell growth inhibition (GI_50_) are summarized in Table 3. The samples of *C. ladanifer* (both from Cerezal and Andévalo origins) exhibited higher potential in all the tested cell lines, presenting GI_50_ values that ranged from 14 to 78 μg/mL in the NCI-H460 and AGS cells, respectively. The sample *J. communis*, presented GI_50_ values of 30.88 ± 1.85 and 41.99 ± 3.60 μg/mL in MCF-7 cell line (from Almazán and Barriomartín, respectively), while *C. sempervirens* showed 20 ± 2 μg/mL in NCI-H460 cell-line (Ermitas origin). In general, all samples showed cytotoxicity in the non-tumor cell lines. However, in the majority of the cell lines, the value of GI_50_ was higher than that of the tumor cell lines, meaning that for particular cases, these EOs can be used without toxicity. Additionally, it can be stated that in vivo studies are needed to verify the toxicity of these oils for specific applications.

All the tested oils showed anti-inflammatory capacity, which is in agreement with Murbach Teles Andrade, Nunes Barbosa, da Silva Probst and Fernandes Júnior [42], who reported that various essential oils exert an anti-inflammatory action by increasing interleukin-10 production. For *J. communis* the plant from Barriomartín showed the best results, but lower than the other species. *C. ladanifer* from Andévalo presented the strongest activity (19 µg/mL), while *C. sempervirens* collected in Ermitas exhibited the highest activity from all the tested samples. Najar, et al. [46] found that *C. ladanifer* EO exhibited cytotoxic activity at 90 ppm for the MCF-7 cell line. However, no further activity was found for the tested cell lines with the essential oils from *J. communis* and *C. sempervirens*.

## 3. Materials and Methods

### 3.1. Plant Material Collection and Conditioning

**Figure 2:**
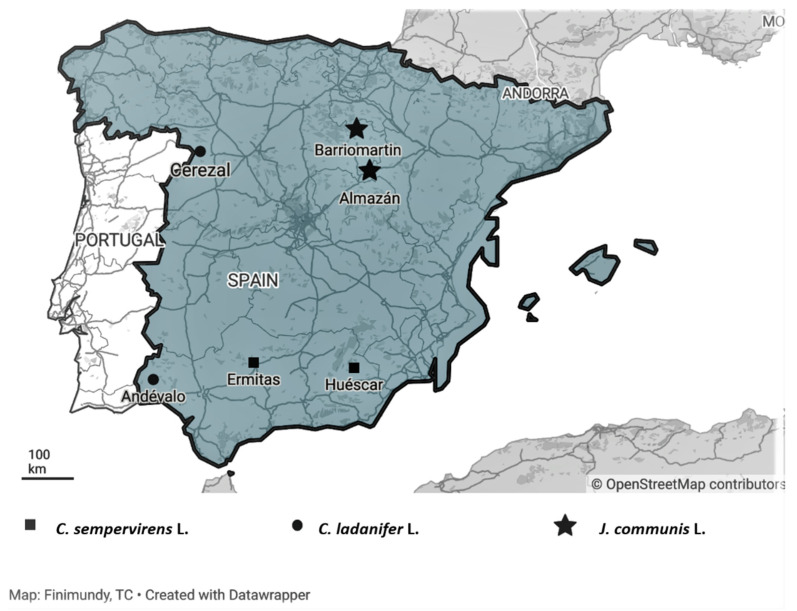
Geographic locations of the collection sites of Spain shrubs populations. Maps generated by Datawapper Online tools.

The plant material (crown biomass, with branches with a maximum stem diameter of 50 mm that included twigs, leaves and fruits) of each one of the species considered was collected in two different locations in Spain: *C. ladanifer* in Cerezal de Aliste and El Andévalo; *C. sempervirens* in Huéscar and Las Ermitas; and *J. communis* in Almazán and Barriomartín (Figure 2 and Table 4).

Samples were randomly taken from a minimum of 10 plants of a similar age, and the biomass of the different plants was mixed to obtain samples of 40 kg of green material from each species.

Previously to the steam distillation, fresh samples were air-dried in the shade at room temperature (10–15 °C) until moisture content was around 10–15%. Afterward, they were ground to a size of 20 mm using a shredder (90 kW, slow rotating single-shaft type, 70 rpm., SILMISA, Onil, Spain). Then, subsamples were taken to determine the moisture content following the standard ISO 18134-2:2017.

### 3.2. Essential Oils Extraction

The ground samples were distilled in a 50 L stainless steel still using steam produced in an electric boiler (ETE, Madrid, Spain). The steam conditions used for the extractions were 13 kg/h with a boiler pressure of 50 kPa. Batch extractions were performed, with two repetitions of 10 kg each per sample and an extraction duration of 2 h for each batch. Time was measured from the moment the first drop of distillate fell. The temperature inside the still was kept constant at 98 °C. The hydrolate and the essential oil were separated by density using a glass Florentine flask. The essential oil samples were then dried using anhydrous sodium sulfate and, after filtration, they were weighed and stored at 4 °C until further analysis. The oil yield for each sample was calculated as a percentage (*w/w*) on a biomass dry weight basis.

### 3.3. Gas Chromatography/Mass Spectrometry (GC-MS) Analyses

The EOs analysis was performed on a GC-MS Perkin Elmer system with a Clarus^®^ 580 GC and a Clarus^®^ SQ 8 S MS module, equipped with DB-5MS fused-silica column (30 m × 0.25 mm i.d., film thickness 0.25 μm; J&W Scientific, Inc., Folsom, CA, USA), according to Falcão, Bacém, Igrejas, Rodrigues, Vilas-Boas and Amaral [45]. The carrier gas was helium gas adjusted to a linear velocity of 30 cm/s. The oven temperature program was as follows: 40 °C for 4 min, raised at 3 °C/min to 175 °C, then at 15 °C/min to 300 °C and held for 10 min. The injector temperature was set at 260 °C, with a transfer line at 280 °C and an ion source at 220 °C. The ionization energy was 70 eV, and a scan range of 35–500 u with a scan time of 0.3 s was used. For each essential oil, 3 µL of sample diluted in HPLC grade n-hexane (1:100) was injected with a split ratio of 1:3. Identification of components was assigned by matching their mass spectra with NIST17 data and by determining the linear retention index (LRI) based on the retention times obtained for a mixture of n-alkanes (C8–C40, ref. 40147-U, Supelco) analyzed under identical conditions. When possible, comparisons were also performed with commercial standard compounds and published data. Quantification was performed using the relative peak area values obtained directly from the total ion current (TIC) values, and the results were expressed as the relative percentage of total volatiles.

### 3.4. Bioactive Evaluation

#### 3.4.1. Antibacterial Activity

To evaluate the antibacterial activity of *C. ladanifer*, *C. sempervirens* and *J. communis* EO, five gram-negative (*Escherichia coli*, *Proteus mirabilis*, *Klebsiella pneumoniae*, *Pseudomonas aeruginosa* and *Morganella morganii*) and three gram-positive bacteria (*Enterococcus faecalis*, *Listeria monocytogenes* and methicillin-resistant *Staphylococcus aureus* (MRSA)) were used. The bacterial strains were clinical isolates obtained from the Northeastern local health unit (Bragança, Portugal) and Hospital Center of Trás-os-Montes and Alto Douro (Vila Real, Portugal). These microorganisms were incubated at 37 °C in an appropriate fresh medium for 24 h before analysis to maintain the exponential growth phase. The antibacterial activity was evaluated through the broth microdilution method, based on the methodology described by Pires, et al. [47], determining the MIC (minimum inhibitory concentration) and the MBC (minimum bactericidal concentration), expressed in mg/mL. Briefly, the samples were serially diluted to obtain the concentration ranges of 2.5 mg/mL to 0.078 mg/mL. Different controls were prepared, namely two negative controls: MHB with Tween 80 and another one with the extract. Two positive controls with MHB with Tween 80 and each inoculum and one culture medium, antibiotics and bacteria. Ampicillin and Imipenem were used for all the tested Gram-negative bacteria and *Listeria monocytogenes*, while ampicillin and vancomycin were selected for *Enterococcus faecalis* and MRSA. After serial dilution in a 96 well microplate, each bacterial inoculum was pipetted to each well (corresponding to 1.5 × 108 Colony Forming Unit (CFU)/mL). The microplates were covered and incubated in a stirring board at 37 °C for 24 h. The MIC values were detected following the addition (50 μL) of 0.2 mg/mL p-iodonitrotetrazolium chloride (INT) and incubation at 37 °C for 30 min. MIC was defined as the lowest concentration that inhibits the visible bacterial growth determined by changing the coloration from yellow to pink if the microorganisms are viable. For the determination of MBC, 10 μL of liquid from each well showed no change in color was plated on solid medium, Blood agar (7% sheep blood) and incubated at 37 °C for 24 h. The lowest concentration that yielded no growth determines the MBC. MBC was defined as the lowest concentration required to kill bacteria.

#### 3.4.2. Antioxidant Activity

The reducing power (RP) Kostić, et al. [48] and cellular antioxidant activity (CAA) [14,49,50] assays were performed to determine the antioxidant potential of the EOs. The reducing power was evaluated by determining the capacity of the extract to reduce Fe^3+^ to Fe^2+^ by measuring the absorbance at 690 nm. The results were expressed as EC_50_ values (μg/mL), corresponding to sample concentration with 0.5 of absorbance. For the CAA, the cells (RAW 264.7) were incubated with different EOs and AAPH (Azobis (2-methylpropionamidine) dihydrochloride), using DCFH-DA (2,7-Dichlorofluorescein diacetate) as a fluorescent marker [14,50]. DCF-DA is a compound that, once in the cell medium, is easily oxidized by peroxide radicals to a fluorescent compound, resulting in DCFH-DA. For the quantification of CAA, the efficacy of the antioxidant treatments was quantified by examining the percentage reduction in fluorescence, according to the formula:

$$\mathrm{CAA}=\%\;\mathrm{reduction}=1-\frac{\mathrm{AUCsample}}{\mathrm{AUCcontrol}}\times 100$$

where AUC sample and AUC control corresponds to the Area Under the Curve of the sample and control, respectively, cells were immediately placed on a microplate reader (FLX800 Biotek, Winooski, VT, USA), where real-time fluorescence was read initially and then every 5 min for 40 min. Fluorescence was measured at an excitation wavelength of 485 nm and an emission wavelength of 538 nm.

#### 3.4.3. Cytotoxicity

To determine the cytotoxic potential of the different EOs, the Sulforhodamine B (SRB) assay [51] was performed on four different human tumor cells obtained from Leibniz-Institut DSMZ-Deutsche Sammlung von Mikroorganismen und Zellkulturen: NCI-H460 (lung carcinoma), MCF-7 (breast carcinoma), AGS (gastric carcinoma) and CaCo (colon carcinoma) and two normal cell lines: PLP2 (porcine liver cells) and VERO (monkey kidney cells). For hepatotoxicity evaluation, the porcine liver primary culture was prepared from a freshly harvested porcine liver. Ellipticine was used as the positive control, and the results were expressed in GI_50_ values (μg/mL), corresponding to the extract concentration that provides 50% of cell growth inhibition [52].

#### 3.4.4. Anti-Inflammatory Activity

The anti-inflammatory activity was determined according to the method formerly reported by Mandim, et al. [53], in which the samples were tested for their capacity to inhibit the lipopolysaccharide (LPS)-induced NO (nitric oxide) production on a murine macrophage cell line (RAW 264.7). Dexamethasone was used as the positive control, and samples without LPS were used as a negative control. The results were expressed as IC_50_ values (µg/mL), corresponding to the extract concentration responsible for 50% of NO production inhibition.

#### 3.4.5. Statistical Analysis

Experimental results were expressed as mean value ± standard deviation (SD). The obtained data were subjected to ANOVA *post-hoc* Tukey’s Honest Significant Difference (HSD) test, applied at *p* < 0.05, using the SPSS v.22.0 program. When less than three repetitions were available, the results were analyzed by the *t*-Student test as a form to determine the significant differences between two samples, with *p* = 0.05.

## 4. Conclusions

The obtained results suggest that the collection of these species in different geographical locations interfere with the essential oil’s yield and respective chemical composition, which can vary in terms of individual compounds’ contents, resulting in different chemotypes. Regardless, no relation was noticeable between the chemical composition and the location’s elevation. Compared with other parts of the plant, in literature, the crown biomass is qualitatively similar in terpenes profile, particularly when taking into consideration the composition specified by the International Organization for Standardization (ISO) and/or the European Pharmacopoeia for the branches and leaves of *C. sempervirens* and *J. communis* berries.

The evaluated species showed to be a viable and low-cost source of EOs that can be used for bio-based products development in different industries, such as the food, cosmetic and medicinal industries. All tested EOs showed the potential to inhibit the growth of gram-negative bacteria, especially *E. coli*, while *C. ladanifer* EO from Cerezal was the only one with the potential to inhibit the growth of *M. morganii*.

Nevertheless, according to the target application, the toxicity exhibited by some of the EOs in the tested tumor cell lines must be deeper analyzed by verifying this condition in specific toxicity models for each industry/product. Therefore, further studies are recommended to deepen the knowledge on these EOs and respective compounds towards different applications.

## Figures and Tables

**Table 1:** Chemical composition of essential oils extracted from tree and shrubs species.

N°	RT (min)	Compound	LRI ^a^	LRI ^b^	Relative % ^c^					
					*C. ladanifer*		*C. sempervirens*		*J. communis*	
					Andévalo	Cerezal	Ermitas	Huéscar	Almazán	Barriomartín
1	8.07	tricyclene	917	921	0.30 ± 0.02	1.08 ± 0.05 ^#^	-	-	-	-
2	8.34	α-thujene	923	924	-	-	0.14 ± 0.01	0.49 ± 0.01 ^#^	0.92 ± 0.01	0.66 ± 0.06
3	8.81	α-pinene	933	932	42.50 ± 0.96 ^#^	19.27 ± 0.26	55.95 ± 0.46	52.32 ± 3.48	23.96 ± 0.41	35.05 ± 0.02 ^#^
4	9.37	α-fenchene	945	945	-	-	0.74 ± 0.03	0.64 ± 0.04	-	-
5	9.42	camphene	946	946	2.15 ± 0.07	6.66 ± 0.01 ^#^	-	-	-	-
6	10.61	sabinene	974	969	-	-	1.07 ± 0.01	2.46 ± 0.20 ^#^	7.86 ± 0.01	6.72 ± 0.40
7	10.70	β-pinene	979	974	-	-	1.25 ± 0.02	1.42 ± 0.13	1.43 ± 0.07	1.63 ± 0.01
8	11.49	β-myrcene	991	988	-	-	2.15 ± 0.07	2.63 ± 0.19	2.60 ± 0.03	3.24 ± 0.06
9	12.08	α-phellandrene	1003	1002	-	-	-	-	1.68 ± 0.06	1.25 ± 0.02
10	12.41	3-carene	1010	1008	-	-	13.09 ± 2.70	16.18 ± 1.12	-	-
11	12.96	p-cymene	1021	1020	2.04 ± 0.01	1.10 ± 0.05	-	-	-	-
12	13.29	limonene	1030	1024	2.07 ± 0.02	1.15 ± 0.09	2.66 ± 0.01	2.74 ± 0.23	21.30 ± 0.03 ^#^	15.01 ± 0.26
13	13.53	trimethylcyclohexanone	1033	1027 *	0.53 ± 0.01	2.57. ± 0.09 ^#^	-	-	-	-
14	14.62	γ-terpinene	1054	1059	-	-	-	-	0.69 ± 0.02	0.50 ± 0.14
15	16.14	terpinolene	1084	1086	-	-	2.60 ± 0.02	3.58 ± 0.34 ^#^	0.94 ± 0.01	0.80 ± 0.03
16	18.64	trans-pinocarveol	1138	1139	1.45 ± 0.05	1.37 ± 0.09	-	-	-	-
17	19.61	pinocarvone	1158	1160	1.01 ± 0.01	1.12 ± 0.02	-	-	-	-
18	20.18	borneol	1170	1165	1.22 ± 0.03	1.05 ± 0.03	-	-	-	-
19	20.57	terpinen-4-ol	1177	1174	-	-	0.12 ± 0.02	0.17 ± 0.03	0.77 ± 0.00	0.47 ± 0.01
20	25.40	bornyl acetate	1282	1283	4.16 ± 0.10	5.01 ± 0.02	-	-	-	-
21	27.99	α-cubebene	1345	1349 *	-	-	-	-	0.51 ± 0.01	0.26 ± 0.01
22	28.15	α-terpinyl acetate	1345	1346	-	-	1.31 ± 0.01	1.33 ± 0.09	-	-
23	28.80	cyclosativene	1360	1369	1.05 ± 0.01	1.24 ± 0.02	-	-	-	-
24	29.15	α-copaene	1368	1374	0.73 ± 0.01	1.15 ± 0.09	-	-	-	-
25	29.85	β-elemene	1384	1389	-	-	-	-	1.48 ± 0.01	1.44 ± 0.01
26	30.98	β-caryophyllene	1411	1408	-	-	-	-	2.14 ± 0.08	3.51 ± 0.14
27	31.65	cis-thujopsene	1427	1429	-	-	-	-	8.19 ± 0.14	8.04 ± 0.07
28	31.70	(E)-thujopsene	1428	1431 *	-	-	-	-	2.63 ± 0.05	1.10 ± 0.01
29	32.47	humulene	1447	1452	-	-	-	-	1.41 ± 0.10 ^#^	0.15 ± 0.04
30	32.65	alloaromadendrene	1451	1455 *	1.54 ± 0.03	2.92 ± 0.05 ^#^	-	-	-	-
31	33.61	germacrene D	1474	1480	-	-	1.38 ± 0.01	3.13 ± 0.19 ^#^	2.69 ± 0.03 ^#^	0.14 ± 0.01
32	33.94	viridiflorene	1482	1496	1.55 ± 0.01	1.50 ± 0.04	-	-	-	-
33	34.62	cuparene	1499	1504	-	-	-	-	0.21 ± 0.01	0.26 ± 0.01
34	34.92	γ-cadinene	1506	1513	-	-	-	-	2.19 ± 0.08 ^#^	0.41 ± 0.01
35	35.13	δ-cadinene	1512	1522	1.25 ± 0.06	1.65 ± 0.03	-	-	1.41 ± 0.01	1.82 ± 0.01
36	36.53	germacrene B	1548	1559	-	-	-	-	0.79 ± 0.04	1.45 ± 0.08 ^#^
37	37.04	palustrol	1561	1567	0.63 ± 0.02	1.06 ± 0.05	-	-	-	-
38	37.44	spathulenol	1571	1577	0.81 ± 0.05	1.69 ± 0.02	-	-	-	-
39	38.39	viridiflorol	1595	1592	13.36 ± 1.14	24.13 ± 0.74 ^#^	-	-	-	-
40	38.58	ledol	1598	1600	4.06 ± 0.17	6.94 ± 0.36	-	-	-	-
41	38.61	cedrol	1601	1602	-	-	2.88 ± 0.11	4.63 ± 0.25 ^#^	1.20 ± 0.12	1.05 ± 0.03
42	38.74	copaborneol	1604	1592 *	1.89 ± 0.03	1.76 ± 0.03	-	-	-	-
43	39.94	τ-muurolol	1647	1640	-	-	-	-	0.25 ± 0.03	0.50 ± 0.15
		**Total Identified**			84.32 ± 0.18	84.48 ± 0.62	85.16 ± 2.17	91.76 ± 0.61	91.91 ± 0.21	88.40 ± 0.31
		**Monoterpene Hydrocarbons**			49.06 ± 1.04 ^#^	29.27 ± 0.27	79.66 ± 2.29	82.48 ± 1.20	61.41 ± 0.24	64.85 ± 0.33
		**Oxygen-Containing Monoterpenes**			7.83 ± 0.09	8.55 ± 0.11	1.43 ± 0.02	1.50 ± 0.12	0.77 ± 0.01	0.47 ± 0.01
		**Sesquiterpene Hydrocarbons**			6.10 ± 0.06	8.47 ± 0.10	1.38 ± 0.01	3.13 ± 0.19 ^#^	26.19 ± 0.38 ^#^	19.30 ± 0.07
		**Oxygen-Containing Sesquiterpenes**			20.78 ± 1.38	35.59 ± 1.00 ^#^	2.88 ± 0.11	4.63 ± 0.25 ^#^	3.53 ± 0.06	3.98 ± 0.04
		**Others**			0.53 ± 0.01	2.57 ± 0.09 ^#^	-	-	-	-

^a^ LRI, linear retention index determined on a DB-5 MS fused silica column relative to a series of n-alkanes (C8–C40). ^b^ linear retention index reported in the literature (Adams, 2017). ^c^ relative % is given as mean ± SD, n = 3. * NIST Standard Reference Database 69: NIST Chemistry WebBook. ^#^ Samples differ significantly (*p* < 0.05) between the different origins, obtained by Student’s *t*-test. Values in bold represent the major compounds present in each sample.

**Table 2:** Antimicrobial activity of the samples obtained by steam distillation (MIC and MBC values; mg/mL).

	Controls																	
	*C. ladanifer*				*C. sempervirens*				*J. communis*				Ampicilin		Imipenem		Vancomycin	
	Andévalo		Cerezal		Ermitas		Huéscar		Almazán		Barriomartín		20 mg/mL		1 mg/mL		1 mg/mL	
Antimicrobial activity	MIC	MBC	MIC	MBC	MIC	MBC	MIC	MBC	MIC	MBC	MIC	MBC	MIC	MBC	MIC	MBC	MIC	MBC
Gram-negative bacteria																		
*Escherichia coli*	0.6	0.6	0.6	0.6	2.5	2.5	2.5	2.5	1.25	1.25	2.5	2.5	<0.15	<0.15	<0.0078	<0.0078	n.t.	n.t.
*Klebsiella pneumoniae*	>2.5	>2.5	>2.5	>2.5	>2.5	>2.5	>2.5	>2.5	>2.5	>2.5	>2.5	>2.5	10	20	<0.0078	<0.0078	n.t.	n.t.
*Morganella morganii*	2.5	2.5	0.6	0.6	>2.5	>2.5	>2.5	>2.5	>2.5	>2.5	>2.5	>2.5	20	>20	<0.0078	<0.0078	n.t.	n.t.
*Proteus mirabilis*	>2.5	>2.5	>2.5	>2.5	>2.5	>2.5	>2.5	>2.5	>2.5	>2.5	>2.5	>2.5	<0.15	<0.15	<0.0078	<0.0078	n.t.	n.t.
*Pseudomonas aeruginosa*	2.5	>2.5	2.5	>2.5	>2.5	>2.5	>2.5	>2.5	>2.5	>2.5	>2.5	>2.5	>20	>20	0.5	1	n.t.	n.t.
Gram-positive bacteria																		
*Enterococcus faecalis*	1.25	1.25	0.6	0.6	2.5	2.5	2.5	2.5	2.5	2.5	2.5	2.5	<0.15	<0.15	n.t.	n.t.	<0.0078	<0.0078
*Listeria monocytogenes*	0.6	0.6	0.3	0.3	2.5	2.5	2.5	2.5	2.5	2.5	>2.5	>2.5	<0.15	<0.15	<0.0078	<0.0078	n.t.	n.t.
MRSA	0.3	0.3	0.07	0.07	2.5	>2.5	2.5	>2.5	2.5	2.5	2.5	2.5	<0.15	<0.15	n.t.	n.t.	0.25	0.25

Essential oils were tested in the concentration range of 2.5 mg/mL to 0.039 mg/mL. n.t. not tested; MIC—minimum inhibitory concentration; MBC—minimum bactericidal concentration. MRSA—methicillin resistant *S. aureus*.

**Table 3:** Antioxidant, cytotoxic and anti-inflammatory activities of *C. ladanifer*, *C. sempervirens*, and *J. communis* essential oils.

	*C. ladanifer*		*C. sempervirens*		*J. communis*		
	Andévalo	Cerezal	Ermitas	Huéscar	Almazán	Barriomartín	Control
Antioxidant Activity							
**Reducing Power Assay (mg/mL)**							Trolox (mg/mL)
EC_50_	1.64 ± 0.34 ^b^	1.30 ± 0.07 ^c^	1.52 ± 0.04 ^b^	1.56 ± 0.01 ^b^	0.97 ± 0.01 ^d^	1.35 ± 0.20 ^b,c^	0.04 ± 0.01
**Cellular Antioxidant Activity (µg/mL)**							Quercetin
% oxidation inhibition *	83.24 ± 2.08 ^a,b^	81.13 ± 3.82 ^a,b^	73.09 ± 2.24 ^c,d^	65.91 ± 1.87 ^e^	78.31 ± 3.08 ^a,b,c^	68.79 ± 3.34 ^d,e^	95.30 ± 4.60 **
GI_50_	336.18 ± 8.09 ^f^	895.45 ± 26.19 ^d^	1196.09 ± 39.41 ^c^	1218.65 ± 18.33 ^c^	324.76 ± 8.13 ^f^	1563.29 ± 58.02 ^a^	0.08 ± 0.01
**Citotoxicity GI_50_ (µg/mL)**							Ellipticine(µg/mL)
AGS	78.41 ± 7.11 ^c^	46.59 ± 4.37 ^d^	260.53 ± 9.25 ^a,b^	289.26 ± 19.61 ^a^	132.68 ± 4.37 ^b^	302.86 ± 7.92 ^a^	1.23 ± 0.03
CaCo2	75.31 ± 0.99 ^c^	48.78 ± 0.09 ^d^	214.28 ± 16.56 ^a,b^	185.98 ± 6.01 ^b^	230.79 ± 5.32 ^a^	107.65 ± 5.15 ^c^	1.21 ± 0.02
MCF-7	27.80 ± 1.28 ^c^	58.45 ± 1.39 ^b^	61.97 ± 5.56 ^b^	165.22 ± 6.95 ^a^	30.88 ± 1.85 ^c^	163.39 ± 5.04 ^a^	1.02 ± 0.02
NCI-H460	14.27 ± 1.31 ^e^	53.80 ± 1.94 ^b^	19.85 ± 1.69 ^d,e^	281.64 ± 16.44 ^a^	44.87 ± 3.42 ^c^	41.99 ± 3.60 ^c^	1.01 ± 0.01
PLP2	207.64 ± 6.44 ^a,b^	142.08 ± 1.60 ^b^	215.18 ± 19.64 ^a^	237.60 ± 25.84 ^a^	241.58 ± 9.52 ^a^	212.03 ± 23.26 ^a^	2.30 ± 0.10
Vero	70.77 ± 4.61 ^c^	46.03 ± 3.60 ^d^	190.95 ± 17.79 ^b^	233.69 ± 21.70 ^a^	240.73 ± 21.32 ^a^	>400	1.10 ± 0.10
**Anti-inflammatory activity IC_50_ (µg/mL)**							Dexamethasone (µg/mL)
IC_50_	19.27 ± 0.37 ^b^	21.00 ± 1.70 ^b^	11.34 ± 1.01 ^c^	14.41 ± 1.27 ^c^	84.80 ± 1.43 ^a^	23.98 ± 0.92 ^b^	6.30 ± 0.40

* at the maximum concentration of 2000 µg/mL. ** Quercetin: % oxidation inhibition: 0.3 µg/mL inhibits 95%. EC_50_: effective concentration at which the absorbance is 0.5 and achieving 50% of antioxidant potential. GI_50_: concentration of the extract causing 50% of cell growth inhibition. IC_50_: concentration providing 50% of inhibition of the NO production in relation to the negative control (100%). Different letters in the same row represent significant difference between means (*p* < 0.05).

**Table 4:** Location and date of the plant material collection in Spain.

Species	Harvesting Place (Province)	Date of Harvesting (DD/MM/YYYY)	Elevation (m)	Coordinates (UTM)
*C. ladanifer*	Andévalo (Huelva)	5 October 2020	199	29T 670374; 4174215
	Cerezal (Zamora)	8 September 2020	828	29T 744316; 4609402
*C. sempervirens*	Ermitas (Córdoba)	17 December 2020	470	30S 339704; 4198106
	Huéscar (Granada)	17 December 2020	988	30S 540991; 4187425
*J. communis*	Almazán (Soria)	14 October 2020	1079	30T 537629; 4601484
	Barriomartín (Soria)	14 September 2020	1402	30T 545081; 4649553

## Data Availability

Not applicable.

## Sample Availability

Samples of the extracts are available from the authors.
